# Integration of graph neural networks and genome-scale metabolic models for predicting gene essentiality

**DOI:** 10.1038/s41540-024-00348-2

**Published:** 2024-03-06

**Authors:** Ramin Hasibi, Tom Michoel, Diego A. Oyarzún

**Affiliations:** 1 Computational Biology Unit, Department of Informatics, University of Bergen, Bergen, Norway; 2 School of Biological Sciences, University of Edinburgh, Edinburgh, UK; 3 School of Informatics, University of Edinburgh, Edinburgh, UK

**Keywords:** Biochemical networks, Cell biology, Computational science

## Abstract

Genome-scale metabolic models are powerful tools for understanding cellular physiology. Flux balance analysis (FBA), in particular, is an optimization-based approach widely employed for predicting metabolic phenotypes. In model microbes such as *Escherichia coli*, FBA has been successful at predicting essential genes, i.e. those genes that impair survival when deleted. A central assumption in this approach is that both wild type and deletion strains optimize the same fitness objective. Although the optimality assumption may hold for the wild type metabolic network, deletion strains are not subject to the same evolutionary pressures and knock-out mutants may steer their metabolism to meet other objectives for survival. Here, we present FlowGAT, a hybrid FBA-machine learning strategy for predicting essentiality directly from wild type metabolic phenotypes. The approach is based on graph-structured representation of metabolic fluxes predicted by FBA, where nodes correspond to enzymatic reactions and edges quantify the propagation of metabolite mass flow between a reaction and its neighbours. We integrate this information into a graph neural network that can be trained on knock-out fitness assay data. Comparisons across different model architectures reveal that FlowGAT predictions for *E. coli* are close to those of FBA for several growth conditions. This suggests that essentiality of enzymatic genes can be predicted by exploiting the inherent network structure of metabolism. Our approach demonstrates the benefits of combining the mechanistic insights afforded by genome-scale models with the ability of deep learning to infer patterns from complex datasets.

## Introduction

The identification of essential genes is crucial for understanding the minimal functional modules required for cell survival^1^, and has key applications in biomedicine and biotechnology^2–4^. For example, essential genes are commonly prioritized as targets for cancer therapy^5^ or as targets for antimicrobial therapies that circumvent resistance mechanisms and improve treatment of severe infections^6^. In industrial biotechnology and metabolic engineering, non-essential genes are normally targeted for knock-down so as to direct metabolic flux away from native processes toward synthesis of high-value products, without compromising cell viability^7^. In general, the identification of essential genes requires screening assays where multiple knock-out mutants are phenotyped with a suitable fitness selection strategy. Such screens have been performed on many organisms, including model microbes such as *Escherichia coli*^8–10^, *Saccharomyces cerevisiae*^11^ and *Bacillus subtilis*^12^, as well as pathogens such as *Candida albicans*^13^ and *Aspergillus fumigatus*^14^. In human cells, recent work has produced high-resolution deletion assays^2^, leveraging progress in high-throughput technologies such as RNA interference and CRISPR-based screens^1^ to produce detailed maps of gene essentiality in different conditions.

As a result of the cost and complexity of knock-out fitness assays, there is a growing interest in computational methods that can complement the experimental work with in silico prediction of fitness effects. These computational approaches often employ machine learning combined with information from protein sequence, gene homologies, gene-function ontologies, and protein interaction networks^15–19^. In the case of metabolic genes, i.e. those that code for catalytic enzymes in metabolic pathways, Flux Balance Analysis (FBA) is a widely employed method for predicting essentiality^20^. There are numerous variants of FBA and its related algorithms^21^, but at its core FBA computes genome-scale flux distributions that optimize a cellular fitness objective. Such objective is typically taken to be the cellular growth rate modeled as a linear combination of synthesis rates of amino acids, lipids and other biomass components. By imposing constraints on each metabolic flux, FBA problems can be solved with efficient linear programming algorithms, which allows to rapidly simulate the impact of gene deletions on the predicted growth rate and draw predictions on the essentiality of metabolic genes.

Flux Balance Analysis has shown good prediction accuracy for gene essentiality in the *E. coli* bacterium^10^ and other model microbes, but predictions for eukaryotes and higher-order organisms have produced mixed results^22,23^. Moreover, the quality of FBA predictions have been shown to vary strongly across different metabolic models and organisms. This caveat is normally ascribed to the quality of the metabolic models themselves, which may contain gaps or errors in the stoichiometry as well as the mapping between enzymatic genes and metabolic reaction^24^. Another often overlooked limitation, however, is the optimality assumption employed by FBA. While there is mounting evidence for optimality of various wild type microbial strains^25,26^, FBA approaches additionally assume that deletion strains optimize the same objective as the wild type. In many cases, however, deletion strains display suboptimal growth phenotypes and are not subject to the same long-term evolutionary pressures as the wild type. It has also been postulated that gene deletions can alter cell physiology to meet other objectives for survival; for example, an early work hypothesized that knockout strains may minimize their phenotypic deviation from the wild type^27^, while various works have explored the impact of alternative objective functions^28,29^ and multiobjective optimization principles^30^ in the classic FBA formulation.

Here, we sought to determine if gene essentiality can be predicted directly from wild type metabolic phenotypes. We developed a hybrid algorithm to predict gene essentiality using a combination of FBA and deep graph neural networks trained on knock-out fitness data. This approach does not require the assumption of optimality of deletion strains and takes maximal advantage of the inherent graph structure of metabolism through the use of a graph neural network as a backbone predictive model. A graph-based model allows including local dependencies between metabolic reactions and its neighbor pathways, and thus improve the ability to predict essentiality of specific metabolic genes. Early attempts to augment the predictive power of FBA with machine learning explored the use of flux features for improved prediction of gene essentiality^31,32^, and other works have attempted to predict essentiality from the metabolic graph topology^33^. Most recently, several authors have developed integrated pipelines aimed at improving FBA predictions for biomedical^34,35^ and biotechnology tasks^36–38^.

In our approach, starting from wild type FBA solutions we first represent genome-scale flux distributions as a weighted digraph in a space of reaction nodes, and employ a flow-based representation for each node based on the redistribution of chemical mass flows between various paths in the graph. To integrate the graph structure and node features into a single predictive model, we employ a Graph Neural Network (GNN) with an attention mechanism^39^ termed FlowGAT. We show that FlowGAT can be trained on a small amount of labelled data from knock-out screens. We demonstrate the effectiveness of our approach using the latest metabolic model of *E. coli*, which achieved a prediction accuracy near the FBA gold standard. Moreover, model predictions appear to generalize well across various growth conditions without the need for further training data. The results highlight the advantages of integrating FBA pipelines with state-of-the-art machine learning algorithms for improved phenotypic predictions.

The structure of our paper is as follows: in Section we detail the core components of the FlowGAT architecture, the graph construction, and our strategy for node featurization. Section presents the various performance evaluation of FlowGAT using data from *E. coli* growing in glucose as carbon source. In Section we explore the ability of FlowGAT to generalize predictions to ten other carbon sources, and we conclude with a discussion on the advantages and caveats of our approach, outlining ideas for future work that could improve FlowGAT and extend its applicability to eukaryotic and higher-order organisms.

## Results

### Model architecture and training

**Fig. 1:**
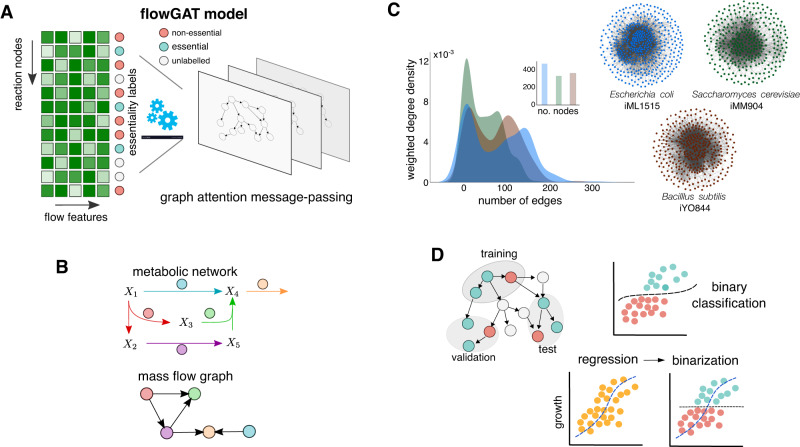
Elements of the FlowGAT model for gene essentiality prediction. **A** Schematic of the FlowGAT architecture proposed in this paper. The model integrates a digraph representation of FBA solutions (Mass Flow Graphs, MFG), where nodes are reactions and edges encode the metabolite mass flows between reactions. We featurize each node with flow-based scores and label them as essential or non-essential using data from gene knock-out assays. Using a graph neural network with an attention layer, FlowGAT predicts essentiality for unlabelled reactions. **B** Construction of mass flow graphs from FBA solutions. The top network is an exemplar metabolic network, and the bottom digraph is the corresponding MFG constructed; nodes are reactions and two nodes are connected if they share metabolites as reactants or products. The edge weights are computed from the metabolite mass flows as described in (3); more details on the MFG construction can be found in Beguerisse-Díaz et al.^42^. **C** Exemplar MFGs for several microbes computed from their genome-scale metabolic models using standard FBA^63^ with the default growth condition in each case; density plots show the distribution of edge weights in each case. **D** For model training and validation, labeled nodes in the MFG are separated into training, validation and test sets. The validation set is used for early stopping and performance metrics are computed on the test set. We explored two training frameworks for FlowGAT: as a binary classifier and as a regressor of growth rate that can be binarized to produce essentiality predictions.

In this paper, we propose FlowGAT, a graph neural network (GNN) model to predict gene essentiality from graphs constructed using FBA solutions. As shown in Fig. 1A, each node in the graph corresponds to a metabolic reaction, and we pair each node with a set of flow-based features and binary essentiality labels obtained from knock-out fitness assays. The graph structure and node features are integrated into a GNN for binary classification, so as to use a message passing scheme to propagate node features through the structure of the graph; this allows learning a rich embedding of the input that contains information from the *k*-hop neighbourhood of each node. According to the message passing algorithm, at each layer of a GNN, each node receives a set of vectors (messages) from its neighbouring nodes and updates its embedding by combining the neighbourhood message set with its embedding vector from previous layer through an aggregation function^40^. The update rule typically contains a combination of message and aggregation functions that can vary depending on the given task and hyperparameters chosen. In our setting, this step is calculated using the attention mechanism, a technique made popular by Transformer architectures^41^. In attention-based message passing, a node learns to focus on the messages that are more informative, and the aggregation function is calculated in a way to highlight the corresponding message effect in the final embedding vector; more details can be found in the Methods. We next detail the different components of the model and our training strategy.

#### Graph construction

We consider metabolic networks with *m* metabolites and *n* enzymatic reactions described by the following differential equation model

1 $$\frac{\,{{\mbox{d}}}X}{{{\mbox{d}}}\,t}={{{\bf{S}}}}v,$$

where *X* is an *m*-dimensional vector of metabolite concentrations, *v* is a *n*-dimensional vector of reaction fluxes, and **S** is a *n* × *m* stoichiometric matrix. In steady state, the relation **S***v* = 0 describes all flux vectors that can sustain a specific metabolic state. A common strategy to estimate *v* at the genome-scale is to employ FBA to compute a flux vector *v*^⋆^ that optimizes a meaningful biological objective; details on FBA can be found in the Methods section. To convert such FBA solution vectors *v*^⋆^ into a graph, we used the Mass Flow Graph (MFG) construction proposed by Beguerisse-Díaz et al.^42^ and illustrated in Fig. 1B. There are numerous ways to convert genome-scale metabolic networks into a graph; for example, some approaches consider nodes as metabolites as nodes and reactions as edges, others consider the opposite and assign reactions as nodes and shared metabolites as edges, or bipartite graphs with both metabolites and reactions as nodes^4^. Among these methods, the MFG construction is well suited for essentiality prediction, because it considers reactions as nodes and therefore the task can be recast a node classification problem using off-the-shelf GNN architectures. Additionally, MFGs account for the directionality of metabolite flow from source to target reactions, as well as the relative weight or contributions of multiple paths for such flows, both of which can improve the predictive power of the models.

Starting from the stoichiometric matrix **S**, we first build a directed graph with reactions as nodes, where two nodes are connected if and only if the source reaction produces a metabolite that is consumed by the target reaction. Each edge in the graph has a weight *w*_*i*,*j*_ that represents the normalized mass flow from node *i* to node *j*. We first compute the flow of metabolite *X*_*k*_ from reaction *i* to *j* according to:

2 $${{{\mbox{Flow}}}}_{i\to j}({X}_{k})={{{\mbox{Flow}}}}_{{R}_{i}}^{+}({X}_{k})\times \frac{{{{\mbox{Flow}}}}_{{R}_{j}}^{-}({X}_{k})}{{\sum }_{\ell \in {C}_{k}}{{{\mbox{Flow}}}\,}_{{R}_{\ell }}^{-}({X}_{k})},$$

where ${\,{{\mbox{Flow}}}\,}_{{R}_{i}}^{+}({X}_{k})$ and ${\,{{\mbox{Flow}}}\,}_{{R}_{j}}^{-}({X}_{k})$ are the production and consumption flows of metabolite *X*_*k*_ by reaction *R*_*i*_ and *R*_*j*_, respectively. The set *C*_*k*_ contains the indices of all reactions that consume metabolite *X*_*k*_. The edge weight *w*_*i*,*j*_ is thus defined as the total mass flow between two nodes, aggregated over all metabolites *X*_*k*_ that are produced by node *i* and consumed by node *j*:

3 $${w}_{i,j}=\mathop{\sum }\limits_{k=1}^{p}{{{\mbox{Flow}}}}_{i\to j}({X}_{k}).$$

Mass flow graphs allow converting FBA solutions into a directed graph, and thus can be used to represent the network structure of metabolism in different growth conditions or genetic perturbations. In Fig. 1C, we show MFGs built from genome-scale metabolic models for three model microbes available in the BiGG model database^43^ (*Escherichia coli*, *Saccharomyces cerevisiae* and *Bacillus subtilis*). Further details on the construction of the mass flow graphs can be found in the Methods section.

#### Design of node features

Besides the graph topology, we ascribe a feature vector to each reaction node that can be exploited for improved performance by the representation learning approach. This approach is analogous to the structural and positional encoding schemes employed in graph Transformer architectures to feed models with extra information about the local connectivity of nodes^44^. Since the edge weights in (3) relate to the directional mass flow between reactions, we opted for features that aggregate information on incoming and outgoing flows from each node. To this end, we employ the Flow Profile Encoding (FPE) first defined by Cooper and Barahona for general directed graphs^45^. Given a directed graph with n nodes and weighted adjacency matrix **A**, for each node *i* we define the inflow profile of length *k* as:

4 $${\,{{\mbox{inflow}}}\,}_{i}^{k}={[{{{{\bf{A}}}}}^{k}{{{{\bf{1}}}}}^{n\times 1}]}_{i},$$

where **A**^*k*^ is the matrix *k*-th power, **1**^*n*×1^ is an *n*-dimensional vector of ones, and [ ⋅ ]_*i*_ is the *i*-th element of a vector. The inflow of node *i* is thus defined as the weighted sum across all incoming paths of length *k*. We similarly define the outflow of node *i* as:

5 $${\,{{\mbox{outflow}}}\,}_{i}^{k}={[{({{{{\bf{A}}}}}^{{\prime} })}^{k}{{{{\bf{1}}}}}^{n\times 1}]}_{i},$$

where ${{{{\bf{A}}}}}^{{\prime} }$ is the matrix transpose. We note that in the case *k* = 1, the definition of inflows and outflows correspond to the in-degree and out-degree of each node, respectively. But when considering longer paths (*k* > 1) the flow profiles describe the pattern of directional flows at longer scales and hence capture higher-order dependencies. We concatenate inflows and outflows up to maximal length *k*_m_ for each node:

6 $$\begin{array}{l}{{{\mbox{FPE}}}}_{i}=\left[{\beta }^{1}\times {\,{{\mbox{inflow}}}\,}_{i}^{1},\cdots \,,{\beta }^{{k}_{{{{\rm{m}}}}}}\times {\,{{\mbox{inflow}}}\,}_{i}^{{k}_{{{{\rm{m}}}}}},{\beta }^{1}\right.\\\left.\qquad\qquad\times {\,{{\mbox{outflow}}}\,}_{i}^{1},\cdots \,,{\beta }^{{k}_{{{{\rm{m}}}}}}\times {\,{{\mbox{outflow}}}\,}_{i}^{{k}_{{{{\rm{m}}}}}}\right],\end{array}$$

where *k*_m_ is a hyperparameter that defines the maximum path length, *β* = *α*/*λ*_1_ is a scaling factor, *λ*_1_ is the largest eigenvalue of the adjacency matrix **A**, and *α* is a hyperparameter that controls for the variable weights of the short and long paths; normalization by the largest eigenvalue *λ*_1_ ensures convergence for large *k*, in the sense that $\mathop{\lim }\nolimits_{k\to \infty }| | {{{{\bf{A}}}}}^{k+1}| | /| | {{{{\bf{A}}}}}^{k}| | ={\lambda }_{1}$. The definition in (6) allows computing a feature vector of length 2*k*_m_ for each node in the graph.

#### Representation learning

Graph representation learning is concerned with mapping the nodes into a low dimensional vector which is optimized for downstream tasks such as classification or regression^46^. GNNs are a family of deep learning methods on graphs which obtain the embedding vector by incorporating the features of the node and its local neighbourhood according to a customized message passing scheme called Message Passing Neural Network (MPNN)^40^. Doing so helps the model capture local and global structural information about the graph and results in a more expressive embedding space. For more information about MPNNs refer to the Methods section. In this study, we employ a MPNN architecture named Graph Attention (GAT) to compute the neighbourhood information importance in finding the representation of the node^39^. Each layer *l* of GAT updates the representation of node *i* according to:

7 $${h}_{i}^{l}=\mathop{\sum}\limits_{j\in {{{\mathcal{N}}}}(i)\cup i}{a}_{ji}{\Theta }^{l}{h}_{j}^{l-1},$$

where ${h}_{i}^{l}$ is the representation vector, ${{{\mathcal{N}}}}(i)$ is the set of neighbouring nodes for node *i*, Θ is a set of differentiable weights, and *a*_*ij*_ is an attention coefficient that is dynamically calculated for each node $j\in {{{\mathcal{N}}}}(i)\cup i$ as

8 $${a}_{ji}=\frac{\exp (\phi ({h}_{i}^{l-1},{h}_{j}^{l-1}))}{{\sum }_{v\in {{{\mathcal{N}}}}(i)\cup i}\exp (\phi ({h}_{i}^{l-1},{h}_{v}^{l-1}))},$$

where *ϕ* is a differentiable function optimized through gradient descent optimization algorithms^47^. Details on the message passing and attention schemes can be found in the Methods section.

#### Data pre-processing

FlowGAT can be trained on knock-out growth assay data, where each gene is labelled as non-essential (0) or essential (1) depending on whether a fitness score is above or below prescribed threshold. For model training, the binary gene labels must be converted into their corresponding reaction node labels in the MFG. To this end, we use the Gene-Protein-Reaction (GPR) map included in genome-scale metabolic models. The GPR is a Boolean function that specifies which gene codes for which proteins, and conversely how each protein affects a metabolic reaction. The GPR can account for reactions that are catalyzed by multiple enzymes or by enzymatic complexes encoded in multiple genes. For those genes that map one-to-one into a single reaction, we transferred the gene label directly into a reaction label. For those genes that map into multiple reactions (many-to-one), we transferred the gene label to all reactions deactivated by the gene deletion. When multiple genes map to multiple reactions (many-to-many), the structure of the GPR does not allow to infer reaction labels from gene labels, and therefore we considered such reactions as unlabelled. Note that the data also contains nodes that lack essentiality labels because their corresponding genes have not been measured in the growth assay. The unlabeled nodes are made available for model training to make sure that the graph representation learning can take advantage of the full graph structure without limiting the representation power of the GAT; the classification loss for training and evaluation of the model is only calculated on the labeled nodes. We also note that the reaction labels are typically imbalanced because the MFG is enriched for essential reaction nodes. By definition in (3), those reactions with zero flux in the wild type FBA solution will have nil edge weights and thus are disconnected from the graph. During training, the model has access to the features of all nodes (labeled and unlabeled) through the message passing, but the training loss is calculated on the labels of the training nodes in semi-supervised fashion (Fig. 1D). Details on model training can be found in the Methods section.

### Performance evaluation

To evaluate the performance of FlowGAT, we employed the growth knock-out data for the *Escherichia coli* bacterium reported by Monk and colleagues^10^, and the iML1515 genome-scale model reported in the same work. We chose the *E. coli* model because it is the most complete and best curated metabolic reconstruction in the literature, and thus allows us to mitigate the impact of misclassification errors caused by poor model quality and focus on the predictive power of FlowGAT itself. The dataset contains growth rate data for 3892 *E. coli* genes in various carbon sources.

**Fig. 2:**
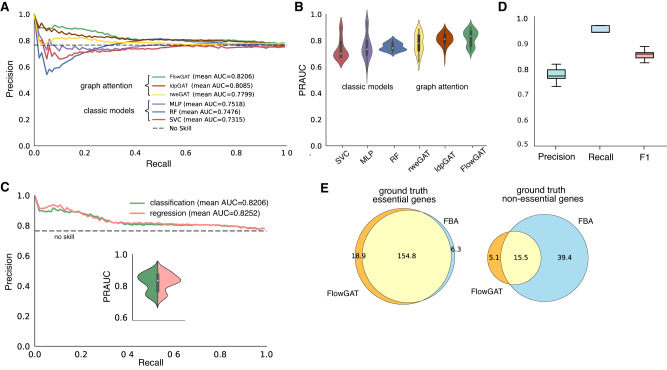
Performance of FlowGAT as predictor of metabolic gene essentiality in *Escherichia coli*. **A** Precision-Recall (PR) curves for classic binary classifiers and graph neural networks trained on essentiality measurements for *E. coli* growing in aerobic conditions with glucose as the sole carbon source^10^. Classic models were trained using the Flow Profile Embeddings (FPE) defined in Eq. (6) computed from wild type FBA solutions. The graph neural networks were trained using the mass flow graph and the FPE as node feature vectors, as well as two other popular node embedding techniques (Local Degree Profile, LDP^49^ and Random Walk Embedding, RWE^50^). Results show PR curves averaged across 50 model evaluations consisting of 5 rounds of testing on 20% test folds and and 10 rounds of model re-training for different random seeds; the dashed line represented the precision (74.9%) of the no-skill classifier given the class imbalance of the data. **B** Distribution of PRAUC scores across the 50 evaluations. The graph attention models outperform classic binary classifiers, and among the three considered node embeddings we found that FlowGAT provides statistically significant increase in performance as compared to traditional models. SVC (*p* = 6.12 × 10^−8^), MLP (*p* = 2.49 × 10^−5^), and RF (*p* = 1.61 × 10^−8^) using a Mann–Whitney *U* test with Bonferroni correction. **C** Retraining FlowGAT as a regressor provides slight gains in performance; inset shows the distribution of PRAUC scores across 50 evaluations; the regressor was trained on growth rate data^10^ and predictions are subsequently binarized to produce essentiality labels. **D** Exemplar classification results by the best performing model (FlowGAT) trained as regressor, as in panel (**C**); box plots show precision, recall and F1-score for the 50 evaluations and a fixed classification threshold; the box shows the median and interquartile range, and whiskers mark the minimum and maximum samples. **E** Comparison between FlowGAT and FBA predictions over the entire gene set in the glucose MFG; Venn diagrams show the number of genes called correctly for each model, averaged across 10 rounds of re-training FlowGAT for different random seeds. Details on model training can be found in the Methods.

We built the MFG for *E. coli* using the wild type FBA solution with glucose as the sole carbon source and the default objective function included in the iML1515 model (growth rate). The resulting MFG has 444 nodes and after converting the gene labels to reaction labels with the GPR map we obtained 255 labeled nodes (191 essential, 64 nonessential). We first compared FlowGAT trained on cross-entropy loss with classical binary classifiers including Support Vector Classifier (SVC), Multi Layer Preceptron (MLP), and random forest (RF) using the flow profile embeddings in (6) as feature vectors; details on model training and hyperparameter selection can be found in Methods. The results in Fig. 2A show precision-recall curves, averaged across *N* = 50 rounds of training and testing (5 test folds with 20% of nodes resampled 10 times for model retraining); details on our strategy for model evaluation can be found in the Methods. Among the considered classifiers, FlowGAT achieves the best Area Under the Precision-Recall Curve (PRAUC) across all test folds and performs above the no-skill classifier while the classic models significantly underperform; we note that due to the class imbalance the baseline precision of the no-skill classifier is 74.9%. The precision-recall curve quantifies the trade off between the false positive and false negative rates, two metrics in binary classification that are particularly important for imbalanced datasets. In the context of gene essentiality prediction, a suitable balance between true positives and true negatives depends on the application at hand. For example, if the aim is to find essential genes that can be targeted to cause cell death^3^, the model should prioritize true positives (high precision). But if the goal is to knockout non-essential pathways to improve production of specific metabolites^48^, the classifier should prioritize true negatives (high recall). The PRAUC score is normally used to find models with a good balance between true positive and true negative predictions, and then the decision threshold is chosen from the curve to achieve a desired performance.

We also compared FlowGAT trained on two other popular node embedding techniques (Local Degree Profile (LDP)^49^ and Random Walk Embedding (RWE)^50^) that have shown good performance in a number of tasks on molecular graphs, as well as two other message passing schemes (Graph Convolutional Network^51^ and GraphSAGE, see Supplementary Fig. 1). Details on these additional node embeddings and message passing strategies can be found in the Methods section. The results (Fig. 2A) show that graph attention delivers the best performance, and models trained on LDP and RWE node features are outperformed by the flow profile encodings, possibly because the former do not take account for directionality and weight of the edges of the MFG.

We further investigated the sensitivity of FlowGAT to the random seed employed for weight initialization; the distributions in Fig. 2B show the PRAUC scores for all models across the 50 runs. The results suggest that FlowGAT performance is relatively robust; only the RF classifier delivers tighter predictions, but at the cost of an average performance below the no-skill baseline.

We finally sought to explore an alternative training scheme using a regression approach. Since the gene essentiality labels are based on a binarization of continuous measurements of growth rate, we reasoned that recasting the prediction problem as a regression task could improve performance. To this end, we employed the non-binarized fitness measurements of growth rate in Monk et al.^10^ and re-trained FlowGAT as a regressor using Mean Squared Error (MSE) loss on predicting the growth rate values; all model hyperparameters were left unchanged. Following the same evaluation scheme as the above, we used the FlowGAT regressor to predict growth rates for the reaction nodes in each test fold. We then used the predicted growth rate as classification scores and computed the precision-recall curve on the test fold. Upon comparison with the classification approach in Fig. 2A, B, the regression results in Fig. 2C led to a performance increase in terms of average PRAUC, as well as tighter predictions that are less sensitive to weight initialization.

After finding the best setting for FlowGAT in terms of architectural design choices, we fixed the cut-off threshold for the output prediction of FlowGAT trained as a regressor in Fig. 2C to produce binary essentiality predictions for all 50 evaluations by classifying the nodes that score above the threshold as essential and others as non-essential. We measure the performance of FlowGAT in terms of three metrics of Precision, Recall, and F1 for the binary predictions (Fig. 2D). The predictions of the FlowGAT regressor manage to keep both precision and recall above 75% and 90%, respectively.

To better understand the performance of our model, we compared the output of FlowGAT trained as a regressor (Fig. 2C) with those from FBA applied to the genes that appear in the MFG. First, we mapped each reaction node back to its corresponding into gene labels using the GPR map. In total, 240 labeled genes appear in the constructed MFG (180 essentials and 60 non-essential). This number is lower than the number of reaction nodes (255) because some reactions correspond to the same gene based on many-to-one mapping that was used to assign labels to nodes earlier; for such genes, we aggregated reactions by maximum prediction value of corresponding reactions. We collected predictions across all genes and compared these results with the essentiality prediction of FBA for each gene in Fig. 2E. The results suggest that both FlowGAT and FBA find most of the essential genes, but FlowGAT finds on average 19 essential genes that are misclassified by FBA. In the case of non-essential genes, however, we found that FlowGAT underperforms and misses more genes than FBA, likely as a result of non-essential genes being the minority class.

### Essentiality prediction in different growth conditions

The essentiality of metabolic genes can be highly dependent on environmental conditions. Different carbon sources produce important changes in metabolic phenotypes and, as a result, some genes that are essential in one condition source may be non-essential in another one.

**Fig. 3:**
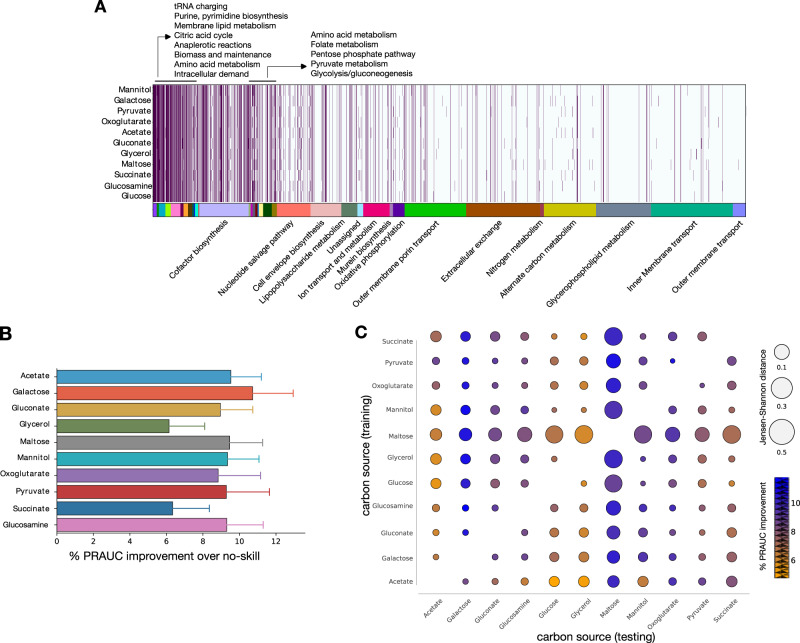
Essentiality predictions of FlowGAT for *Escherichia coli* growing in different carbon sources. **A** Heatmap of reactions present in mass flow graphs (MFG) computed from wild type FBA solutions computed for ten carbon sources. Each MFG is obtained through changing the carbon source; the color bar denotes the different metabolic subsystems as annotated in the latest genome-scale metabolic model iML1515^10^. **B** Prediction performance of FlowGAT trained and tested on the different condition-dependent MFGs. Bars show the average improvement of PRAUC scores across the 50 evaluations with respect to the no-skill classifier; error bars denote one standard deviation of the PRAUC. Full precision-recall curves for each case can be found in Supplementary Fig. 2. **C** Performance of FlowGAT in cross-testing across different carbon sources; in each case, the model was trained on one graph and tested on the nodes of all other graphs, totalling 90 cross-test evaluations. The color bar indicates the improvement in PRAUC over the no-skill classifier; the bubble radius denotes the graph-to-graph distance computed as the Jansen-Shannon divergence between the distribution of node features. In panels (**B**, **C**), the performance improvement was computed as $100\times \left({{{\mbox{PRAUC}}}}_{{{{\rm{FlowGAT}}}}}-{{{\mbox{PRAUC}}}}_{{{{\rm{no}}}}{{\mbox{-}}}{{{\rm{skill}}}}}\right)/{{{\mbox{PRAUC}}}}_{{{{\rm{no}}}}{{\mbox{-}}}{{{\rm{skill}}}}}$.

To test the predictive power of FlowGAT in other carbon sources beyond glucose, we trained the model using *E. coli* knock-out fitness data in ten other carbon sources that cover different entry points into central carbon metabolism^10^. We built the corresponding mass flow graphs from wild type FBA solutions of the iML1515 model instanced to each carbon source. To build condition-dependent graphs, we constrained the flux of each nutrient exchange reaction to a fixed value (Supplementary Table 2). This resulted in 10 different MFGs that differ on their nodes and their edge weights. Inspection of the reaction nodes per graph (Fig. 3A) reveals differences across graphs for reactions that become active for specific carbon sources, as well as a large number of reactions that are shared across conditions. We then evaluated the performance of FlowGAT trained in each of the ten mass flow graphs, using the growth knock-out fitness data and the regression strategy of Fig. 2C; model hyperparameters were left unchanged. In each graph, the number of essential and non-essential nodes varies and thus, the no-skill baseline varies depending on the class imbalance in that graph. As seen in Fig. 3B, we found that while the PRAUC scores vary across growth conditions, in all cases FlowGAT outperformed the no-skill classifier by at least 6%. These encouraging results can likely still be improved by introducing condition-specific hyperparameters for the FlowGAT architecture.

We finally aimed to determine the ability of FlowGAT to generalize predictions across growth conditions. We conducted a cross-training evaluation, where the model was trained on a mass flow graph and fitness data from a single carbon source, and tested on the reaction nodes in a different growth condition. To this end, we also included the FlowGAT model for glucose discussed in the previous section. As shown in Fig. 3C, all 90 cross-tests show an improvement in PRAUC with respect to each MFG no-skill classifier. Although each FlowGAT model was trained on a different graph and fitness data, these results suggest that the model captures a well-performing representation of the data. To test if this is a result of the similarity between the nodes present in each graph (Fig. 3A), we quantified the graph-to-graph similarity using the distance between the distribution of node features. We estimated the probability density function of the flow profile encodings for each graph using kernel density estimation and computed the Jensen-Shannon divergence between all pairs of distributions. The results (Fig. 3C) do not show a correlation between graph similarity and the PRAUC scores. For example, the maltose graph embedding is nearly equidistant from both the acetate and galactose graphs, but FlowGAT trained on maltose has a performance of approximately 5% better when tested on galactose than in acetate. Likewise, FlowGAT trained on mannitol performs better when tested in galactose than glycerol, despite the galactose graph being more dissimilar to the mannitol graph. These observations suggest that the generalization performance of FlowGAT results from its representation power rather than the similarity between the input graphs.

## Discussion

Essential genes often encode proteins that play critical roles in cellular processes needed for growth. Since the quantification of gene essentiality requires knock-out fitness assays across a large number of genes and growth conditions, there is substantial interest in computational methods that can aid the identification of genes from a reduced number of measurements. In this paper, we presented FlowGAT, a graph neural network that can be trained on knock-out fitness data to predict the essentiality of metabolic genes. The architecture exploits the inherent graph structure of metabolic fluxes predicted by Flux Balance Analysis through a combination of mass flow graphs and node features that describe local connectivity.

Using data from *E. coli* and its latest genome-scale metabolic model, we show that FlowGAT can identify most of the genes that are correctly called as essential by Flux Balance Analysis, and even correct some of its misclassified essential genes. Our approach is based solely on the wild type phenotype predicted by FBA; since it does not require the assumption of optimality of deletion strains, FlowGAT may be suitable for other microbes where gene deletions lead to suboptimal growth. In the case of higher-order organisms and cell types, FlowGAT holds promise for cases in which optimality of the wild type optimality can be assumed, such as some cancer types that are amenable to FBA analyses^23^. We also note that the construction of mass flow graphs does not require the flux vector to be optimal. This opens the possibility of extending FlowGAT to cases in which the optimality assumption fails even for the wild type. For example by replacing the FBA step with other descriptions of the flux space, such as elementary flux modes, extreme pathways or flux sampling^21^.

Additionally, we observed an encouraging generalization power of FlowGAT across growth conditions, even in cases where the underlying graphs and node features differ substantially. This suggests that the proposed architecture and feature extraction method can learn internal representations that are useful predictors of gene essentiality. Accurate prediction of essentiality across conditions can potentially reduce cost and efforts in experimental essentiality screens, and lead to testable hypotheses on genetic liabilities that emerge in specific cellular environments. While this approach holds great promise, we recognize the inherent challenge of predicting gene essentiality in different contexts due to its variable nature across cell types and growth conditions^5^. Future approaches will likely require training data the combine fitness data across multiple cellular contexts, so as to improve the quality of predictions.

We also found that FlowGAT struggled to predict non-essential genes and can be outperformed by traditional FBA. This phenomenon could result from non-essential genes being intrinsically more challenging to predict, or from the strong class imbalance that is implicit in the FlowGAT approach. By construction, mass flow graphs are enriched for essential reaction nodes, because zero flux reactions led to disconnected nodes. As a result, the class imbalance favors essential labels at the detriment of poor predictions for non-essential genes. The implications of this poor performance depend on the end application; for example, if the aim is to discover druggable targets against pathogens^3^, the focus is on accurately detecting essential genes that can be inhibited and cause cell death. Conversely, in metabolic engineering applications, the focus is on detecting non-essential genes that can be safely knocked down and alleviate their competition with a target production pathway^48^. Further extensions to our work could address the class imbalance with data augmentation techniques or using class-specific penalization in the loss function employed for training.

The integration of artificial intelligence and machine learning algorithms into various biological disciplines is advancing at a rapid pace and has found applications in many domains such as strain design^52,53^, drug discovery^54^ and metabolic engineering^55^. Our approach illustrates the potential of combining well-adopted tools such as Flux Balance Analysis with modern data-driven approaches, and adds to the growing body of literature^36,56–58^ at the interface of metabolic modelling and machine learning.

## Methods

### Flux balance analysis

Flux Balance Analysis (FBA) is one of the popular methods for the analysis of cellular metabolism. In a steady state, a metabolic network can be described by

9 $${{{\bf{S}}}}v=0.$$

The aim of FBA is to obtain the solution vector v^*^ that satisfies the above condition and at the same time solves the following optimization problem:

10 $$\begin{array}{ll}{v}^{*}=\arg \mathop{\max}\limits_{v}\quad{c}^{{\prime} }v\\\quad\,\,\, {{{\rm{subject}}}}\,{{{\rm{to}}}}\quad\left\{\begin{array}{l}{{{\bf{S}}}}v=0,\quad \\ {v}_{{{{\rm{lb}}}}}\, < \,v\, < \,{v}_{{{{\rm{ub}}}}},\quad \end{array}\right.\end{array}$$

in which, *c* is a vector of flux weights, and (*v*_lb_, *v*_ub_) are lower and upper bounds on reaction fluxes, respectively.

### Mass flow graphs

Originally introduced by Beguerisse-Díaz et al.^42^, Mass Flow Graphs (MFGs) are designed to reflect the directional flow of metabolites produced or consumed through enzymatic reactions. In these graphs, reactions are considered as vertices, and two reactions are connected through a directed edge if they share a metabolite (either as reactants or products). The construction pipeline of these graphs can incorporate different experimental conditions through varying flux distributions.

To construct an MFG from a metabolite network consisting of *m* reactions and *n* metabolites, first we obtain the solution vector v* from FBA. Then, we unfold the v* into two-fold forward and reverse reaction fluxes through

11 $${v}_{2m}^{* }=\frac{1}{2}\left[\begin{array}{c}\,{{\mbox{abs}}}\,({v}^{* })+{v}^{* }\\ \,{{\mbox{abs}}}\,({v}^{* })-{v}^{* }\\ \end{array}\right].$$

Next, the corresponding stoichiometric matrix of ${v}_{2m}^{* }$ is defined as

12 $${{{{\bf{S}}}}}_{2m}=\left[\begin{array}{cc}{{{\bf{S}}}}&-{{{\bf{S}}}}\end{array}\right]\left[\begin{array}{cc}{{{{\bf{I}}}}}_{m}&0\\ 0&\,{{\mbox{diag}}}\,(r)\\ \end{array}\right],$$

in which, S in the *n* × *m* stoichiometric matrix corresponds to *n* reactions and *m* metabolites of the original network, and *r* is an *m* dimensional Boolean vector indicating whether a reaction is reversible or not. Finally, the adjacency matrix of the MFG can be calculated as

13 $$A({v}^{* })={({{{{\bf{S}}}}}_{2m}^{+}{{{{\bf{V}}}}}^{* })}^{{\prime} }{{{{\bf{J}}}}}_{v}^{{\dagger} }({{{{\bf{S}}}}}_{2m}^{-}{{{{\bf{V}}}}}^{* }),$$

where † is the matrix pseudoinverse operator, and ${{{{\bf{V}}}}}^{*}=\,{{\mbox{diag}}}\,({v}_{2m}^{*})$, ${{{\bf{J}}}}=\,{{\mbox{diag}}}\,({{{{\bf{S}}}}}_{2m}^{+}{v}_{2m}^{* })$ with

14 $${{{{\bf{S}}}}}_{2m}^{+}=\frac{1}{2}(\,{{\mbox{abs}}}\,({{{{\bf{S}}}}}_{2m})+{{{{\bf{S}}}}}_{2m}),$$

15 $${{{{\bf{S}}}}}_{2m}^{-}=\frac{1}{2}(\,{{\mbox{abs}}}\,({{{{\bf{S}}}}}_{2m})-{{{{\bf{S}}}}}_{2m}).$$

### Node feature generation

Mass Flow Graphs do not include features for each node (reactions). As a result, it is necessary to design a feature generation pipeline that considers the structure of the graph as well as the edge weights that appear in the adjacency of the graph. For this task, we propose a node encoding algorithm analogous to positional encoding of Transformer architectures.

Apart from the proposed FPE features in Eq. (6), a second approach to node encoding is to gather local neighborhood structural statistics based on the degree of each node and its neighboring nodes^49^. In this approach, the local degree profile of each node is defined as

16 $${{{\mbox{LDP}}}}_{i}=[\,{{\mbox{deg}}}(i),\min ({{\mbox{DN}}}(i)),\max ({{\mbox{DN}}}(i)),{{\mbox{avg}}}({{\mbox{DN}}}(i)),{{\mbox{std}}}({{\mbox{DN}}}\,(i))],$$

in which DN(*i*) is the set of out-degree values for all the neighboring nodes of node *i*. The minimum, maximum, average, and standard deviation of the out-degree are calculated and used as node features of node *i*. Additionally, a third encoding method relies on random walks from each node. In this method, a random walk encoding for each node *i* is calculated as:

17 $${{{\mbox{RWE}}}}_{i}=[R{W}_{ii}^{2},R{W}_{ii}^{3},\ldots ,R{W}_{ii}^{{K}_{\max }}],$$

where ${K}_{\max }$ is a hyperparameter for maximum length of the random walks, and ***RW*** = **AD**^−1^ is the random walk operator and only the random walks that end in node *i* are considered for encoding (*RW*_*ii*_ is this the *i*-th element of the diagonal).

### Message-passing neural networks (MPNN)

For representation learning of the graph features, we employed the Graph Attention (GAT) architecture^39^ which is an instance of a MPNN scheme. In a typical graph representation learning task, the representation of each node is updated through a message passing scheme in which the information from neighboring nodes is gathered using message formula and aggregated with the features of the node itself. Thus, a message passing formula for each message from node *j* to node *i* can be written as

18 $${m}_{ji}^{(l)}={{{\mbox{MSG}}}}^{(l)}\left({h}_{j\in \{{{{\mathcal{N}}}}(i)\cup i\}}^{(l-1)},{e}_{j,i}\right)$$

where ${h}_{i}^{(l)}$ is the representation vector of node *i* in layer (*l*) of the MPNN, *e*_*j*,*i*_ are the features of the edge between node *i* and node *j*, and ${{{\mathcal{N}}}}(i)$ is the set of neighbouring nodes to node *i*. The operator MSG^*l*^ is the custom message function which is different in each layer design. One typical example of such a function is an MLP applied on the input values. Moreover, the messages for each node are aggregated to obtain the representation of node *i* in layer *l* using

19 $${h}_{i}^{(l)}={{{\mbox{AGG}}}}^{(l)}\left(\left\{{m}_{ji}^{(l)},u\in {{{\mathcal{N}}}}(i)\right\},{h}_{v}^{(l-1)}\right),$$

in which, AGG is a custom permutation invariant operator with regards to messages for each node.

Graph Attention formulates the message equation in (18) as the multiplication of the attention as the learnable importance factor of each message by the representation of neighbors. Thus, the formula in (18) becomes:

20 $${m}_{ji}^{(l)}={a}_{ji}{\Theta }^{l}{h}_{j}^{l-1},$$

in which, *a*_*ji*_ is the attention coefficient and is usually calculated through feeding the features of both neighboring nodes *i* and *j* through a learnable function and calculating the importance through the softmax function. Other popular examples of MPNN framework are Graph Convolution Network (GCN)^51^ and GraphSAGE^59^ which change the message function and use different aggregation functions. In GCN, the message function in (18) is calculated as:

21 $${m}_{ji}^{l}=\frac{{\Theta }^{l}{h}_{j}^{l-1}}{\sqrt{\,{{\mbox{deg}}}(i)}\sqrt{{{\mbox{deg}}}\,(j)}},$$

with the sum pooling operator as the aggregator function. In GraphSAGE, the message function MSG is the identity function and the aggregation function AGG is calculated as:

22 $${h}_{i}^{l}={\Theta }^{l}{h}_{i}^{l-1}+{\Theta }^{{\prime} l}{{{{\rm{MEAN}}}}}_{j\in {{{\mathcal{N}}}}(i)}{h}_{j}^{l-1}.$$

where MEAN is the mean pooling operator. The comparison between the performance of different MPNN schemes is presented in the Supplementary Figure 1.

### Graph construction

To build the MFGs, we employed the iML1515 model of *E. coli* MG1655 introduced by Monk et al.^10^. To label the reaction nodes in the graph, we employed the growth assay data from the same work on strain BW25113. Since BW25113 lacks several genes from MG1655, we produced FBA solutions by setting their reaction bounds to zero and assuming aerobic growth. The reaction bounds can be found in Supplementary Table 1. To simulate *E. coli* growth in specific carbon sources, we set the corresponding exchange flux to a fixed value and deactivated all other carbon exchange fluxes. The list of all carbon sources and their corresponding exchange reactions can be found in Supplementary Table 2. All calculations were done with the COBRApy toolbox v0.26.3 using the glpk solver and the default objective function included in the iML1515 model.

### Performance evaluation of binary classifiers

#### Training and evaluation in a single carbon source

We started our evaluations of FlowGAT from the MFG computed with glucose as the sole carbon source in Fig. 2A. After mapping reactions to genes based on GPR rule set, growth rate values were converted to essentiality labels based on the threshold of 0.5 and were assigned to corresponding nodes in the MFG. The labeled nodes in the MFG are imbalanced with a higher number of essentials compared to non-essential nodes. Therefore, for model training, we employed stratified sampling into 5 folds using built-in scikit-learn^60^ functions with 1 fold for testing and 4 folds for training with the labeled nodes and 25% of the training set is set chosen as validation set (Fig. 1D). For the initial tuning of hyperparameters, we employed grid search for each model and chose the best model settings based on the performance on the validation set. We performed a grid search and trained the model using many hyperparameter combinations. The trained model was then evaluated on the validation set and a negative log likelihood loss value was computed. After collecting the loss value for all possible combinations, the hyperparameters set that achieved the minimal loss were chosen as the final configuration; these hyperparameters were kept constant for all other evaluations in the paper. All GNN models were implemented in the PyG package^61^, while training and hyperparameter tuning were done with GraphGym; classic models (SVC, MLP, RF) were implemented using scikit-learn. The space for the grid search and final hyperparameters for each model can be found in Supplementary Tables S3, S4.

Due to the small number of labeled genes available in our dataset (255 in case of glucose MFG), to compare the performance of different models (Fig. 2) we trained the GNN models on the training folds and evaluated the performance on the test fold 5 times, each time changing the train and test fold to ensure that the results are not caused by split bias. In the case of GNN based models, for each evaluation step, 25% of the training fold was considered as the early stopping set. We kept track of the best model on the early stopping set, in terms of the loss value after each training epoch, until the maximum number of epochs was reached. The weights of the best model at the end of training were then saved and employed to predict for the nodes of the test fold. Additionally, each training and evaluation step on a test fold was repeated 10 times with the model retrained with a different initial random seed to make sure the predictions were not a result of random seed selection for weight initialization. In total, 50 evaluation steps (5 folds and 10 times for each fold) were gathered for each model. For the classic models (SVC, RF, MLP), we employed the same process except for the use early stopping set. We followed the same procedure for model evaluation in other carbon sources (Fig. 3B).

#### Training and evaluations across carbon sources

To produce the evaluations in Fig. 3C, for each MFG the training and early stopping folds were chosen with a 4:1 ratio; in all cases we tested each model on all nodes of the other MFGs. Following the same scheme as in the previous section, the best performing model on the early stopping set was chosen for the evaluation of the test set; the training set was resampled 5 times and each model was retrained 10 times with different initial weights.

### Reporting summary

Further information on research design is available in the Nature Research Reporting Summary linked to this article.

### Supplementary information

Supplemental Material

Reporting summary

Supplementary Data File

## Data Availability

Gene essentiality predictions for FBA and FlowGAT using the iML1515 model for *Escherichia coli* can be found in the Supplementary Data file, alongside the ground truth labels from Monk et al.^10^.

## Code availability

Python code for model training and evaluation is available in Zenodo^62^ at 10.5281/zenodo.10551318.
