# Supplementary material for: Integration of graph neural networks and genome-scale metabolic models for predicting gene essentiality
Source: NPJ Syst Biol Appl. 2024 Mar 6;10:24. doi: 10.1038/s41540-024-00348-2 (PMC10917767; doi:10.1038/s41540-024-00348-2)
Supplement: Supplementary file 1 — Supplemental Material [file 41540_2024_348_MOESM1_ESM.pdf]

Supplementary Figures and Tables

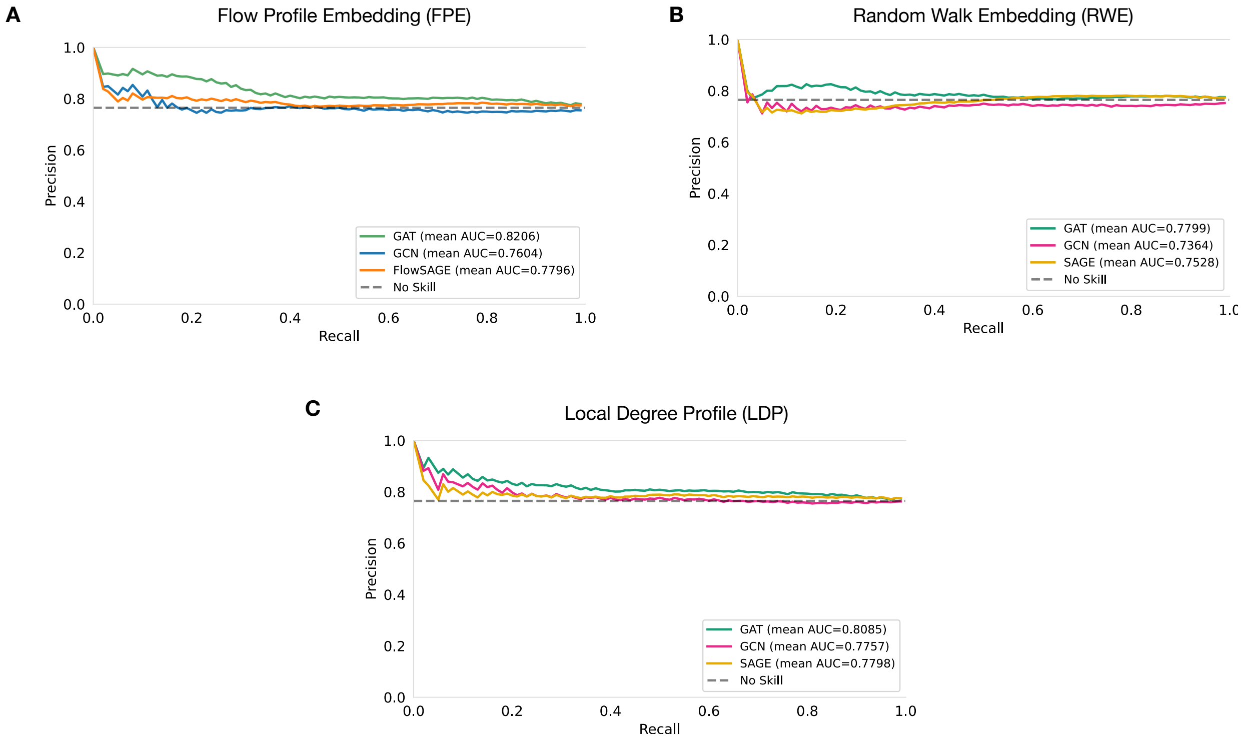

Supplementary Figure 1. **Performance comparison between different message passing schemes and node embeddings.** Model training and evaluation followed the same procedure as Figure 2A in the main text. Panels (**A**–**C**) show results show three node embeddings: Flow Profile Embeddings defined in Eq. (6), Random Walk Embeddings, and Local Degree Profile as explained in the Methods, as well as three popular message passing schemes (Graph Attention as in Figure 2A, Graph Convolution Networks, and Graph SAGE).

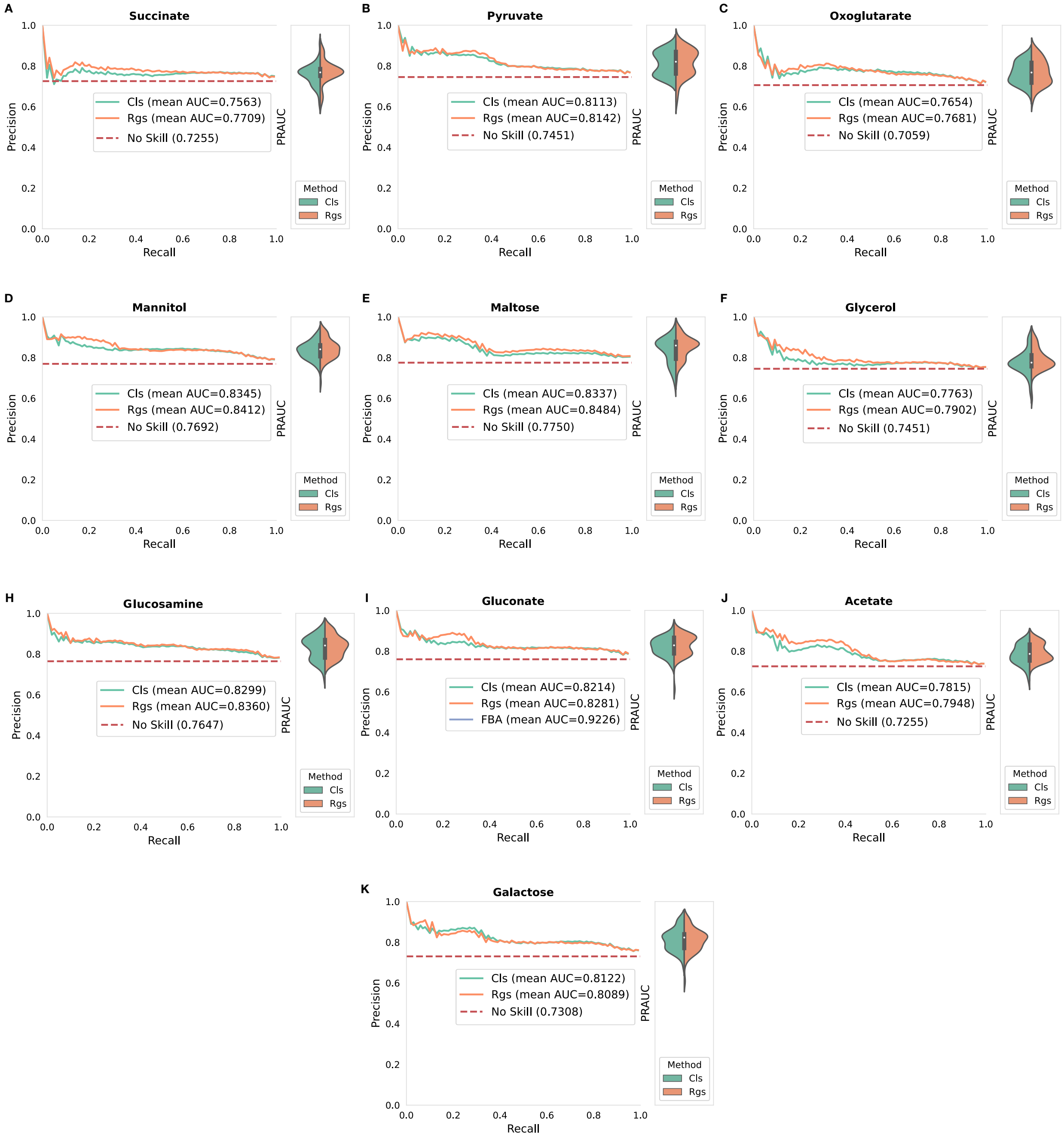

Supplementary Figure 2. **Performance comparison of classification and regression training for FlowGAT trained on labels and mass flow graphs for various carbon sources.** For each of the ten graphs, FlowGAT was trained on the nodes of the graph using both classification and regression training scheme, as in Figure 2C in the main text. Precision-recall curves were averaged over 5 cross-validation folds and 10 rounds of re-training for different random seeds. Panels (**A**–**K**) show results for the 10 carbon sources considered in the main text. The boxplots show the median and interquartile range of the distribution, and whiskers show the minimum and maximum samples.

Reaction	Bounds
L-arabinose isomerase (ARAI)<br>L-ribulokinase (RBK_L1)<br>Rhamnulose-1-phosphate aldolase (RMPA)<br>Lyxose isomerase (LYXI)<br>L-rhamnose isomerase (RMI),<br>Rhamnulokinase (RMK)<br>B-galactosidase (LACZ)	$v_{lb} = v_{ub} = 0$
Oxygen exchange (EX_o2_e)	$v_{lb} = -20$

Supplementary Table 1. **Bounds for exchange reactions for the *E. coli* iML1515 model growing in aerobic conditions with glucose as the only carbon source.**

Carbon source	Reaction	Bounds
Succinate	EX_succ_e	$v_{lb} = -10$
Pyruvate	EX_pyr_e	
Oxoglutarate	EX_akg_e	
Mannitol	EX_mnl_e	
Maltose	EX_malt_e	
Glycerol	EX_glyc_e	
Glucosamine	EX_acgam_e	
Gluconate	EX_glcn_e	
Acetate	EX_ac_e	
Galactose	EX_gal_e	

Supplementary Table 2. **Exchange reactions for different carbon sources in the *E. coli* iML1515 genome-scale metabolic model.** For each carbon source, the corresponding reaction bounds were adjusted and all other reaction bounds for other sources were set to 0.

	Hyperparameter	Search space	Final value
Architecture	Number of message passing layers	[2, 4]	4
	Number of post message passing layers	[1, 2]	1
	Number of pre-message passing layers	[0, 1, 2]	0
	Dimension of hidden layers	[8, 16, 32]	16
	Activation function	[ReLU]	ReLU
	Dropout rate	[0.1, 0.3]	0.1
	k_{m}	[4, 8]	8
Training	Base learning rate	-	0.01
	Learning rate decay	-	0.1
	Momentum	-	0.9
	Optimization algorithm	-	ADAM
	Scheduler	-	Cosine Annealing
	Max epochs	-	200
	Min epochs	-	20
	Classification loss	-	Cross entropy
	Regression loss	-	MSE

Supplementary Table 3. **Hyperparameters list for Graph Neural Network (GNN) models.** For each GNN the final value for the list of above hyperparameters was determined after initial tuning on a chosen validation set through grid search. Tuning was done on architectural hyperparameters and the suggested set by GraphGym was reused for training hyperparameters. The final hyperparameter values were used for all evaluations reported in the paper. For a fair comparison among different GNNs, the architecture and training hyperparameters were kept unchanged and the message passing formula is the only change in the architecture (e.g., GAT, GCN, SAGE)

Model	Hyperparameter	Search space	Final value
RF	Criterion	[gini, entropy]	entropy
	Max depth	[40, 50, 60]	50
	Max features	[sqrt]	sqrt
	Num estimators	[100, 200, 300]	300
SVC	Kernel	[poly, rbf]	rbf
	Gamma	[scale, auto]	scale
MLP	Activation function	[ReLU, Sigmoid]	ReLU
	Optimizer	[ADAM]	ADAM
	Hidden layer size	[80, 100, 120]	100
	Number of layers	[2, 3, 4]	2

Supplementary Table 4. **Hyperparameters list for classic machine learning models in Figure 2A–B.** Hyperparameters were determined after initial tuning on a chosen validation set through grid search over the indicated space.
